# Privacy-Aware Collaborative Learning for Skin Cancer Prediction

**DOI:** 10.3390/diagnostics13132264

**Published:** 2023-07-04

**Authors:** Qurat ul Ain, Muhammad Amir Khan, Muhammad Mateen Yaqoob, Umar Farooq Khattak, Zohaib Sajid, Muhammad Ijaz Khan, Amal Al-Rasheed

**Affiliations:** 1 Department of Computer Science, COMSATS University Islamabad, Abbottabad Campus, Abbottabad 22060, Pakistan; annietariq44@gmail.com (Q.u.A.); mateen@cuiatd.edu.pk (M.M.Y.); 2 School of Information Technology, UNITAR International University, Kelana Jaya, Petaling Jaya 47301, Selangor, Malaysia; 3 Computer Science Department, Faculty of Computer Sciences, ILMA University, Karachi 75190, Pakistan; zohaib.sajid@ilmauniversity.edu.pk; 4 Institute of Computing and Information Technology, Gomal University, Dera Ismail Khan 29220, Pakistan; ijaz171@gmail.com; 5 Department of Information Systems, College of Computer and Information Sciences, Princess Nourah bint Abdulrahman University, Riyadh 11671, Saudi Arabia; aaalrasheed@pnu.edu.sa

**Keywords:** federated learning, skin cancer classification, SVM, neural networks, privacy-aware learning

## Abstract

Cancer, including the highly dangerous melanoma, is marked by uncontrolled cell growth and the possibility of spreading to other parts of the body. However, the conventional approach to machine learning relies on centralized training data, posing challenges for data privacy in healthcare systems driven by artificial intelligence. The collection of data from diverse sensors leads to increased computing costs, while privacy restrictions make it challenging to employ traditional machine learning methods. Researchers are currently confronted with the formidable task of developing a skin cancer prediction technique that takes privacy concerns into account while simultaneously improving accuracy. In this work, we aimed to propose a decentralized privacy-aware learning mechanism to accurately predict melanoma skin cancer. In this research we analyzed federated learning from the skin cancer database. The results from the study showed that 92% accuracy was achieved by the proposed method, which was higher than baseline algorithms.

## 1. Introduction

**Figure 1:**
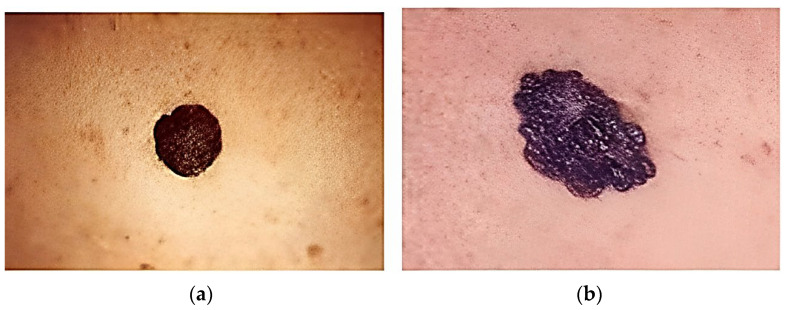
Types of melanoma skin cancers. (**a**) Benign; (**b**) malignant.

Health encompasses the overall well-being of an individual, encompassing mental, physical, and social aspects that contribute to a higher quality of life. However, global health faces challenges arising from factors such as limited healthcare resources, disparities between rural and urban areas, and inadequate services. Early detection plays a crucial role in the effective treatment of skin cancer, a particularly serious form of the disease. The skin acts as a protective barrier for internal organs and structures, emphasizing the significant impact that even minor disruptions can have on the body’s systems. The diverse nature of skin disorders is reflected in the varying appearance and severity of skin lesions. The many types of skin cells that contribute to the development of each lesion are used to categorize them. Melanocytes, the cells responsible for producing the protein pigment melanin, are damaged or destroyed in melanocytic diseases such as melanoma [1]. Types of melanoma skin cancer are shown in Figure 1.

Melanoma, the deadliest type of skin cancer among approximately 200 different types, can be diagnosed through clinical screening, dermoscopy, and histology [2]. Early detection significantly increases the chances of successful treatment. Dermoscopy images, when combined with visual inspection by specialists, improve the accuracy of melanoma diagnosis, raising it from 65–80% to 75%. Mobile edge computing (MEC) offers computational work offloading and low-latency services, which are particularly valuable for time-sensitive applications. However, optimizing reliability and latency in MEC remains a challenging task. Data privacy concerns have led to the establishment of regulations such as the GDPR [3], China Cyber Security Law [4], and CCPA [5], granting individuals rights pertaining to data collection, disclosure, erasure, and protection against automated decision making. The CCPA specifically applies to for-profit businesses operating in California, ensuring transparency in data handling.

**Figure 2:**
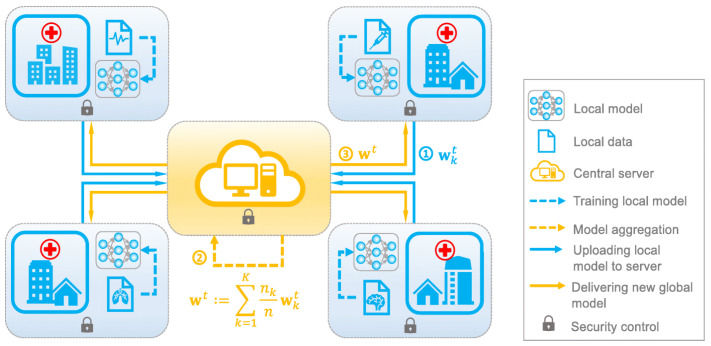
Federated learning.

Leveraging data from multiple institutions can be advantageous in addressing class imbalance and expanding databases. However, sharing medical records faces obstacles due to privacy, technical, and regulatory limitations [6]. Google’s federated learning approach aims to train machine learning algorithms on local datasets without explicit data sharing [7]. Local nodes exchange parameters to build a common global model capable of accommodating diverse datasets and unreliable clients, as shown in Figure 2. In centralized federated learning, a central server oversees participating nodes and algorithmic stages. Decentralized federated learning, on the other hand, avoids a single point of failure by exchanging model updates across interconnected nodes without relying on a central server. This approach prevents potential bottlenecks caused by update transmission. By integrating federated learning and AI in a hybrid strategy, healthcare systems can navigate restrictions and privacy concerns while training models in a distributed manner.

The data privacy problem was the main motivation to solve these highlighted issues. This article presents a novel approach for optimizing communication in federated learning by utilizing an asynchronous strategy for updating the parameters of shallow and deep layers of deep neural networks (DNNs) at different frequencies [8,9,10,11,12,13]. The proposed method aims to reduce the amount of communication required between the server and clients, which can be a significant bottleneck in federated learning. Additionally, the article suggests a temporally weighted aggregation strategy to effectively incorporate the information from previously trained local models into the final model aggregation. This approach allows the model to effectively learn from the unique characteristics of each local dataset and improve learning performance overall. The proposed method aimed to achieve a balance between communication efficiency and learning performance, making it a valuable approach for implementing federated learning in real-world scenarios.

The major contributions of our work are listed as follows.

- We proposed an optimal classification for skin cancer detection.
- To improve the communication concern, we used asynchronous for skin cancer.
- We improved the convergence rate by using FL.
- We achieved more accuracy by using these methods.

## 2. Related Work

The purpose of this review was to assess the present status of research on machine learning-based techniques for skin cancer detection and classification. It investigated the application of various algorithms, datasets, assessment measures, and field problems, providing insights into advancements and future approaches for better skin cancer diagnosis. The authors introduced an improved method for classifying skin cancer in [14]. Their approach utilized deep convolutional neural networks (CNNs) with transfer learning, demonstrating its effectiveness in achieving accurate classification results by leveraging pre-trained models and fine-tuning them on skin cancer datasets.

The authors in [15] addressed the issue of shortcut learning in machine-learning models used for skin lesion diagnosis. They identified the presence of shortcut learning in these models, which can lead to misleading results. Their work aimed to enhance the reliability and accuracy of machine learning models for skin cancer diagnosis. A comparison of different machine-learning strategies for analyzing infrared thermography images in skin cancer detection was outlined in [16]. They evaluated the performance of various algorithms and discussed the advantages and limitations of each approach. The study provided insights into selecting appropriate machine-learning techniques for infrared thermography-based skin cancer diagnosis.

The paper [17] presented a machine learning-based approach for predicting skin conditions. The authors proposed dynamic training and testing augmentation techniques to improve prediction accuracy. Their study demonstrated the effectiveness of this approach in accurately predicting skin conditions based on various input features. A framework focused on region-of-interest (ROI) and transfer learning was proposed in [18]. They aimed to enhance the efficiency and accuracy of skin cancer detection by considering specific regions within the images. Their results showed the effectiveness of the proposed framework in achieving reliable skin cancer detection results.

The authors in [19] highlighted the advantages of combining human expertise with artificial intelligence (AI) for skin cancer classification. The authors emphasized the importance of leveraging the knowledge and expertise of dermatologists alongside AI algorithms to improve the accuracy and reliability of skin cancer classification systems. The authors in [20] addressed obstacles and future possibilities by discussing various ML approaches used for skin cancer detection and classification. The research highlighted the potential of machine learning in improving skin cancer diagnostic outcomes.

The authors in [21,22,23,24,25,26,27,28,29,30,31] discussed several elements of skin cancer detection and classification using machine learning. They also looked at the use of deep convolutional neural networks, transfer learning models, infrared thermography, dynamic training and testing augmentation, region-of-interest-based transfer learning, and the combination of human and artificial intelligence in skin cancer detection, classification, and prediction. The current benchmark models for skin cancer detection are only available to non-compliant healthcare providers. As a result, the focus of this study was on constructing a privacy-preserving model for melanoma skin cancer datasets to assess the efficacy of the suggested strategy in terms of accuracy.

## 3. Materials and Methods

**Figure 3:**
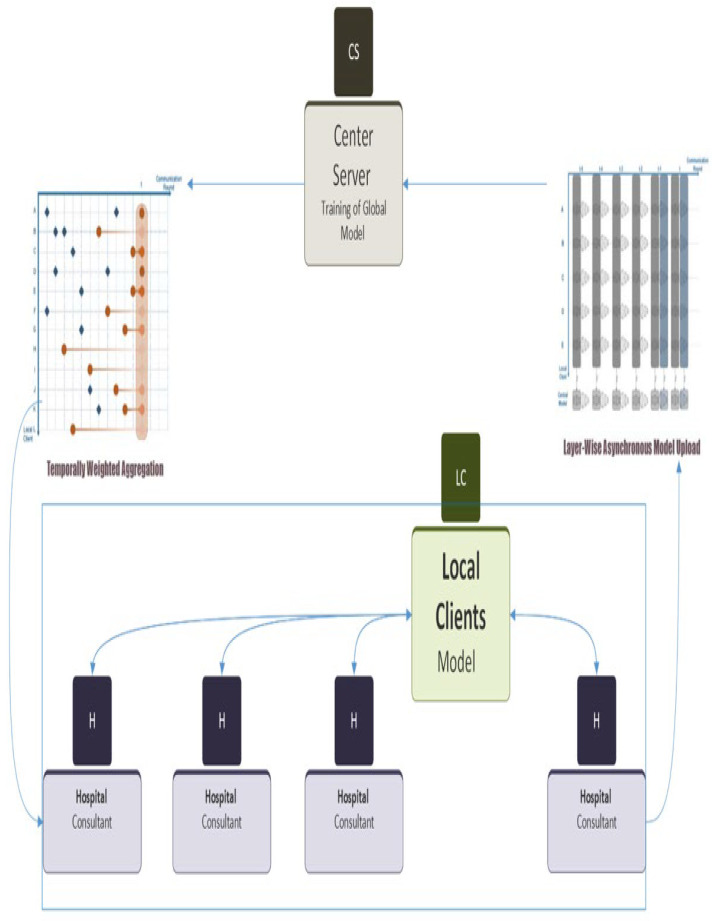
Proposed Methodology Using Federated Learning.

The existing healthcare system faces several data privacy issues [6,7] and the existing ML techniques [8,9,10,11,12,13,14,15,16,17,18,19,20,21,22,23,24,25,26,27,28,29,30] are unable to address privacy concerns since they require user data to be processed at a central location for model generation and skin cancer diagnosis. User data cannot be shared in the healthcare system owing to privacy and security concerns. This research proposed a federated learning framework for melanoma skin cancer prediction in the healthcare system, which would solve privacy issues and deliver effective skin cancer prediction in a privacy-aware healthcare system. This present study sought to explore the feasibility and efficacy of federated learning in a healthcare context, with the goal of creating machine learning models that would be both accurate and secure. In Figure 3, the proposed methodology involved leveraging the collective computing power of multiple hospitals to train models that would be representative of the patient population at each facility. The following steps were taken to accomplish this objective:

- **Selection of Four Hospitals**: The study involved the participation of four hospitals, chosen based on their capacity to contribute data to the project and their willingness to collaborate. The hospitals were chosen to ensure geographic diversity and representation of both public and private healthcare facilities.
- **Local Training using SVM and CNN:** The data collected from each hospital was preprocessed and used to train support vector machines (SVMs) and convolutional neural networks (CNNs) locally. This step ensured that each hospital’s data were used to create models that were specific to their patient population, thereby enhancing model accuracy.
- **Conversion of Data into Weights:** Following the completion of local training, the data were transformed into weight values that were representative of the learned patterns within the models. This process ensured that only model parameters were exchanged between hospitals, preserving data privacy and security.
- **Transfer of Local Weights to Cloud for Training:** The weight values obtained from the individual hospitals were then transferred to a cloud-based server for further training. The central server aggregated the weight values and used them to update a global model, which incorporated the latest learnings from all participating hospitals.
- **Federated Learning using Asynchronous Method:** The global model was then used to perform federated learning using an asynchronous method. This approach enabled the central server to train the model using the updated weights from each hospital, without the need for synchronous communication, which reduced the communication overhead and latency between clients and server.
- **Distribution of Updated Weights to Clients:** The updated weight values were sent back to each hospital according to their request. This step allowed each hospital to incorporate the latest learnings from the global model into their locally trained models, thereby improving model accuracy and generalization performance. The distribution of these weights is done asynchronously as depicted in Figure 3. The different colored weights shows weights at different time.

The proposed methodology offered several benefits, including enhanced data privacy and security, reduced communication overhead, and improved model accuracy. By enabling hospitals to train models locally and contribute to a shared global model, the approach offered a scalable and sustainable means of creating machine learning models that would be representative of diverse patient populations. This study showcased the potential of federated learning in a healthcare context and suggested that it may offer a viable means of creating models that would be both accurate and secure. The working of our proposed Async-FL model for both client and the server ends is discussed in Algorithm 1 below.
**Algorithm 1.** Proposed Async-FL model for skin cancer predictionInput: Skin Lesion data from various client users Output: Personalized models for skin cancer prediction **//Working at the FL server end** for round r = 1, 2, … do if r in every round_i n_loop ∈ Set _ES_ then§  tag ← 1 else  tag ← 0 max← maximum(A∗B.1) t_s_ ← (random set of maxclients) every client j є _ts_ in parallel do if tag then  w^j^ ← ClientUpdate(j, ws,tag)  timeframe_k_^g^ ← s  timeframe_k_^s^ ← s else  w_k_^g^ ← ClientUpdate(j, w_k,s_, tag)  timeframe_k_^g^ ← s  $w_{k,s+1}\;\leftarrow \;\sum_{g}^{b}=1\frac{m_{g}}{m}$ *fk(s,j)*w_k_^g^
if tag then  $w_{t,s+1}\;\leftarrow \;\sum_{g}^{b}=1\frac{m_{g}}{m}$*ft (s,j)*w_k_^g^
end function **//Working at FL client** client update (j, w, tag)//client j α ← (fragment b_j_ into batches of size A) if tag then       w ← w else      w_t_ ← w for local epoch j from 1 to f do for batch a є α do        perform classification using radial basis SVM if tag then       return w to server else return w_t_ to server end function

### 3.1. Rules for Skin Lesion Assessment

The proposed system is a technique for the identification and categorization of skin cancer through the utilization of imagery. The process begins by transmitting an image to the system, which is then subject to preprocessing utilizing the median filtering method to eliminate extraneous elements such as hair, bubbles, and noise. This method is employed as the skin cancer image often contains fine hair, noise, and bubbles, which are not indicative of the presence of cancer and therefore must be removed. The median filter is chosen for its ability to preserve the amplitude and location of edges while also reducing the variance of intensities in the image, resulting in a smoothing effect on the overall image. After preprocessing, the preprocessed image is segmented using the watershed algorithm. The watershed algorithm is a popular image segmentation method that partitions an image into multiple segments or regions. It is based on the idea that the image can be seen as a topographic surface, where the intensity of the pixels represents the height of the surface. The algorithm starts by identifying the local minima of the image, which are used as markers for the initial segmentation. Then, it propagates the markers to the surrounding pixels, creating a catchment basin for each marker. The result is a set of segments where each segment corresponds to a catchment basin. The GLCM feature technique, ABCD rule, and shape feature are then used to extract features from the photos. The texture characteristics of the lesion are extracted using the GLCM feature technique. The statistical technique known as GLCM, or gray-level co-occurrence matrix, defines the texture of an image by calculating the likelihood that certain gray-level combinations would appear in the picture. Shape feature is utilized to extract the shapes such as irregularity index, abnormality index, and distance that are evaluated from lesions in the binary picture. Lastly, classification approaches are employed to achieve the best outcomes. To achieve these best outcomes, the system employs three different categorization methods. The ABCD rule and GLCM feature technique are the basis for the first kind of classification and the shape feature is the basis for the second type of classification; the combination of all the features forms the basis for the third type of classification. By comparing the outcomes of each classification technique and choosing the one with the highest accuracy, the system employs these three forms of classification to arrive at the best results.

### 3.2. Experimentation Details and Dataset Utilized

Our experimentation did not involve any human subjects, instead we utilized the images of skin lesions. For evaluation and testing of our proposed model, we utilized the publicly available dataset of International Skin Imaging Collaboration (ISIC-2019) dataset, which contains 25,331 dermoscopy images. In this study, we considered only the melanoma skin cancer type. For the experimentation, simulation, and performance analysis, we used the *PyTorch* machine learning library. The experimentation trials were carried out using the NVIDIA Tesla T4 16 GB graphics processing unit (GPU) on Google Colaboratory.

## 4. Results and Discussion

The proposed system is a privacy-preserving decentralized learning method for recognizing and classifying melanoma skin cancer using an image. It uses a combination of preprocessing, segmentation, feature extraction, and classification techniques to identify the characteristics of the lesion and to classify it as malignant or benign.

### 4.1. Experimental Results of the Proposed Model

**Figure 4:**
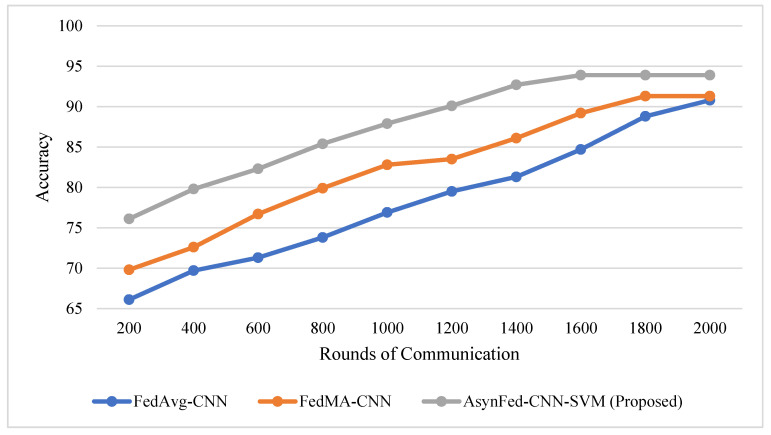
Effect of convergence rate on accuracy.

In this section, a detailed comparison of proposed Async-Fed-CNN-SVM is made with the existing state-of-the-art federated learning methods. The rate at which a learning algorithm approaches its optimal solution, known as the convergence rate, can impact the overall accuracy of the solution. A fast convergence rate may lead to a solution quickly, but it does not guarantee a higher level of accuracy. On the other hand, a slower convergence rate may take longer to reach a solution but may provide a more accurate outcome. The relationship between convergence rate and accuracy can vary depending on the specific algorithm and dataset being used, as depicted in Figure 4. Generally, a fast convergence rate is preferred, but it must be considered in relation to potential problems, such as overfitting. Our proposed Async-Fed-CNN-SVM uses a better classification and training model at the client end, which results in better accuracy achievement in a lesser number of communication rounds. 

### 4.2. Effect of Learning Rate in Training

**Figure 5:**
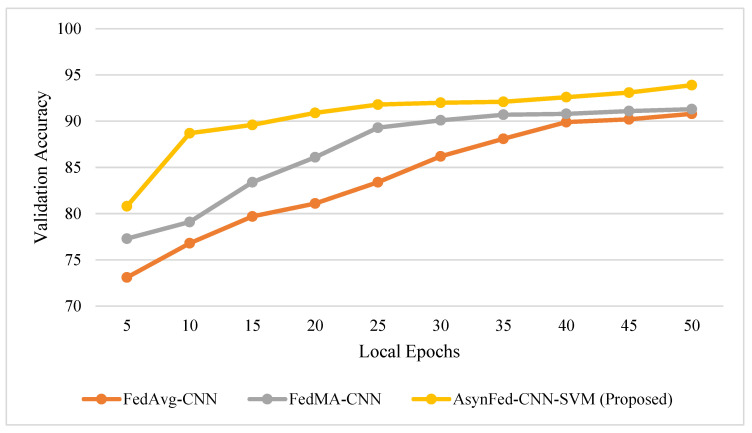
Effect of learning rate on validation.

The learning rate is a key parameter in machine learning that determines the step size of updates made to the model’s weights during training and is shown below in Figure 5 and Table 1. The choice of learning rate can have a significant impact on the performance of the model. A low learning rate will lead to slow convergence but more accurate results, while a high learning rate will lead to faster convergence but less accurate results. It is common to use a moderate learning rate initially and adjust it based on the performance of the model.

The ABCD rule is a tool used to identify potential melanomas (a type of skin cancer) by looking at the asymmetry, border, color, and diameter of a lesion and Table 2 shows the ABCD rule-based performance comparison. The gray-level co-occurrence matrix (GLCM) is a technique used to extract texture information from an image and the performance analysis based on GLCM rule is shown in Table 3. It creates a matrix that describes the relationship between pixels of different gray levels and is often used in image analysis and computer vision. The learning rate is an important parameter in machine learning that controls the step size at which the model updates its parameters. It determines how quickly or slowly the model learns from the data. A smaller learning rate will lead to a more accurate model, but it will take longer to train. A larger learning rate will lead to faster training, but the model may not be as accurate. Finding the right balance between these two extremes is crucial for successful training. Table 4 shows the maximum achieved performance by our proposed method.

## 5. Conclusions

In this study, we investigated an approach for identifying and categorizing melanoma skin cancer using a federated learning method. Our suggested approach relied on using a segmentation method to separate the lesion from the skin and a classification method to identify the lesion’s malignancy or benignity. We used the hybrid of CNN with SVM technique for the prediction and classification of melanoma skin cancer. We used the ABCD rule, which assesses skin lesions based on certain criteria, to support the vector machine (SVM) method for classification. We contrasted SVM’s performance with that of other classifiers such as random forest and KNN. Our results demonstrated that our proposed Async-Fed-CNN-SVM achieved 92% accuracy, which was better than the other assessed federated learning methods. Even with biased training samples, SVM is recognized for its accuracy and robustness. Furthermore, because SVM’s optimality problem is convex, it provides a special solution. The threshold for differentiating between benign and malignant lesions can be chosen with some flexibility. Additionally, it exhibits good generalization to out-of-sample data. This locally operated nonparametric function allows for a nonlinear threshold that can change depending on the data’s features. Overall, by proposing a method that combines CNN with SVM in the federated learning environment appearing as the most potent classifier, our research has contributed to the field of skin cancer detection and classification.

## Figures and Tables

**Table 1:** Learning rate of our proposed method.

No. of Epochs	5	10	15	20	25	30	35	40	45	50
Accuracy training	81.1	88.8	89.7	91.1	92.0	92.3	92.4	92.8	93.6	94.7
Validation accuracy	80.8	88.7	89.6	90.9	91.8	92.0	92.1	92.6	93.1	93.9

**Table 2:** ABCD rule-based analysis of performance analysis.

Parameters	Name of the Classifier		
	SVM (Proposed)	KNN	Random Forest
Sensitivity (%)	90.1	76.9	77.1
Specificity (%)	89.1	72.7	79.9
Accuracy (%)	92.1	69.1	76.8

**Table 3:** GLCM feature-based performance analysis.

Parameters	Name of Classifier		
	SVM (Proposed)	KNN	Random Forest
Sensitivity	86.1	65.8	74.9
Specificity (%)	87.1	68.7	77.2
Accuracy (%)	88.1	63.1	77.1

**Table 4:** Maximum achieved accuracy and loss.

Training	94%
Validation	94%
Testing	93%
Training loss	3%
Validation loss	4%
Testing loss	5%

## Data Availability

We ran simulations to see how well the proposed approach performed. Any questions concerning the study in this publication are welcome and can be directed to the lead author (Qurat ul Ain) upon request.
